# Application of Molecular Hydrogen in Early Heart Failure Development: Modulation of Microcirculation, Metabolism, Oxidative Stress, and Myocardial Status

**DOI:** 10.3390/antiox14121418

**Published:** 2025-11-27

**Authors:** Anna Vyacheslavovna Deryugina, Darya Andreevna Danilova, Anastasia Vladimirovna Polozova

**Affiliations:** Department of Physiology and Anatomy, Institute of Biology and Biomedicine, National Research Lobachevsky State University of Nizhny Novgorod, 23 Prospekt Gagarina (Gagarin Avenue), Nizhny Novgorod 603950, Russia; p0lozovaav@yandex.ru

**Keywords:** molecular hydrogen (H_2_), microcirculation, lipid peroxidation, tissue perfusion

## Abstract

Oxidative stress is a key factor in the development of chronic heart failure (CHF). Molecular hydrogen (H_2_) exhibits antioxidant properties, yet the mechanisms by which it alleviates hemodynamic disturbances and ischemic myocardial injury in CHF are not fully understood. This study examined the effects of a single (40-min) and multiple (40-min daily for 5 days) inhalations of H_2_ in a rat model of CHF induced by catecholamine administration. Microcirculatory function was evaluated using laser Doppler flowmetry and laser fluorescence spectroscopy. Lipid peroxidation levels in plasma and myocardium were measured, and histological analysis of myocardial tissue was performed. The findings demonstrated that H_2_ inhalation improved microvascular perfusion (*p* < 0.05) by activating local regulation and restoring central control mechanisms. This contrasts with the decreased perfusion and disrupted adaptive regulation observed in CHF. Notably, oxidative stress and metabolic abnormalities induced in the model were significantly mitigated by H_2_, with the most substantial effects observed after multiple administrations (*p* < 0.05). Histological assessments revealed that repeated H_2_ inhalation reduces myocardial edema and preserves tissue morphology during cardiac remodeling. In conclusion, hydrogen therapy shows potential for delaying CHF progression at early stages by normalizing microcirculation and tissue metabolism.

## 1. Introduction

Currently, cardiovascular diseases are the leading cause of death and disability worldwide [1]. According to the World Health Organization, more than 16 million people die annually from these diseases. One of the most common progressive and prognostically unfavorable conditions of the cardiovascular system is chronic heart failure (CHF). The prevalence of CHF in developed countries is approximately 1–2% among the general population and between 10% and 25% among individuals over 65 years old [2]. Despite the availability of a wide range of modern medications that can achieve positive outcomes in the treatment of CHF, there is an unwavering increase in the prevalence of this pathology across all population groups [1]. During the first five years after the onset of clinical symptoms of heart failure, 75% of men and over 60% of women die [3]. The high prevalence and mortality rates underscore the need to identify effective and safe compounds, along with an analysis of their molecular and cellular mechanisms of action at early, preclinical stages of heart failure development. This approach aims to improve life expectancy and quality of life, as well as to reduce the costs of treatment.

Heart failure arises from various myocardial and non-myocardial factors [4]. Myocardial causes include hypoxia, infections, toxins, metabolic disorders, vitamin deficiencies, and mechanical or electrical injuries [5,6]. Non-myocardial causes involve valvular disease, hypertensive crises, anemia, stenosis, and endocardial fibrosis [7,8]. These factors disturb intracardiac and systemic hemodynamics. They also induce damaging processes such as ischemia/reperfusion injury, inflammation, oxidative stress, and endothelial dysfunction. Oxidative stress (OS) plays a key role in the pathogenesis and progression of cardiovascular disease. Excessive production of reactive oxygen species (ROS) exerts a detrimental effect on proteins, lipids, and DNA in all cells of the body, including cardiomyocytes [9]. Free radicals damage cardiomyocytes, leading to myocardial remodeling and impaired contractile function. They also induce endothelial dysfunction, resulting in increased vasoconstriction and hypercoagulability, and contribute to metabolic and energetic imbalances in tissues. Considering the critical role of reactive oxygen species (ROS) in the pathogenesis of heart failure, it is crucial to develop effective antioxidant strategies to prevent the progression of chronic heart failure. An important approach involves early correction of the body’s condition to limit the development of oxidative stress and reduce ROS levels to physiological ranges, thereby restoring cellular homeostasis and stabilizing cardiac function. At present, there is no universally effective and safe method for treating and preventing diseases associated with oxidative stress due to dysregulated free radicals. The use of many clinically validated antioxidants is limited to a narrow therapeutic dose range due to their high toxicity levels, which consequently renders preventive strategies against oxidative stress-related diseases ineffective.

From the perspective of emerging antioxidant strategies, molecular hydrogen (H_2_) is gaining increasing attention as a promising therapeutic agent. To date, its protective and therapeutic effects have been demonstrated in various pathologies, including neurodegenerative diseases [10], cardiovascular conditions [11], hematological disorders [12], sepsis [13], metabolic diseases, and respiratory illnesses [14]. H_2_ exhibits selective antioxidant activity. It specifically neutralizes reactive oxygen species (ROS) and reactive nitrogen species (RNS), such as ^•^OH and ONOO^−^. H_2_ does not affect superoxide anion radical or other peroxides involved in normal physiological processes [15].

Besides its direct antioxidant activity, H_2_ has been shown to participate in antiapoptotic, anti-inflammatory, and cytoprotective effects [16]. Currently, H_2_ is often referred to as a gaseous signaling molecule [17], similar to NO, CO, and hydrogen sulfide (H_2_S), from both physiological and therapeutic perspectives [18]. Moreover, H_2_ offers several advantages for clinical application: it exhibits no toxic properties [19] and readily penetrates biological barriers through gas diffusion [20]. Inhalation of gaseous H_2_ is considered the most effective method of delivering molecular hydrogen to the body. Other administration routes include oral intake of hydrogen-rich water, injections of hydrogen-saline solutions, and direct diffusion of molecular hydrogen, which is present in eye drops, baths, and cosmetic products [21].

Despite the significant potential of H_2_, its effect on myocardial metabolism during the early stages of heart failure development remains inadequately studied. Since myocardial dysfunction is directly related to the status of the microcirculatory system, understanding how H_2_ influences the maintenance of adequate tissue perfusion is of critical importance.

In this context, we previously investigated the antioxidant effects of H_2_ in an experimental model of chronic heart failure in rats. Our results demonstrated H_2_′s ability to improve blood rheological properties by reducing erythrocyte aggregation, increasing electrophoretic mobility, and preserving optimal morphological and functional characteristics of erythrocytes. Additionally, H_2_ decreased the levels of secondary lipid peroxidation products within erythrocytes, thereby enabling them to fully perform their gas-transporting function [22].

Building on our previous findings, this study aims to further elucidate the therapeutic potential and primary mechanisms of H_2_ in the early stages of heart failure development. The results provide compelling evidence that, through its antioxidant effects, H_2_ supports adequate myocardial tissue perfusion by regulating microcirculation and metabolic processes, thereby preventing the formation of irreversible structural damages that impair cardiac tissue function. We anticipate that a deeper understanding of the interactions between oxidative stress and the antioxidant actions of H_2_ may inspire new approaches for improving therapeutic strategies for chronic heart failure.

In light of the above, the objective of this study was to investigate the effects of H_2_ on microcirculation, tissue and oxidative metabolism, and the morpho-functional state of the heart during the early stages of chronic heart failure development in a rat model.

## 2. Materials and Methods

### 2.1. Animal Model and Care

This study was approved by the Local Ethical Committee for Animal Research at National Research Lobachevsky State University of Nizhny Novgorod on 9 October 2020, №40 and was conducted in accordance with the guidelines of the European Community (EU Directive 2010/63/EU, 2010). Male Wistar rats (*n* = 40), weighing 260 ± 20 g, were obtained from the vivarium of Lobachevsky University (Nizhny Novgorod, Russia). The animals were acclimated for one week prior to experimentation. They were housed under standard laboratory conditions with a 12 h light/dark cycle, temperature of 23 ± 3 °C, and humidity of 50–60%. Food and water were provided ad libitum. The study was performed in accordance with the principles for animal research outlined in the ARRIVE 2.0 guidelines [23].

### 2.2. Verification of CHF Rats Model

Using rats is appropriate because their cardiovascular systems resemble humans, and the heart failure model mimics key human features, enabling relevant studies for understanding and treating human chronic heart failure (CHF). In this study, a total of 40 animals were used, of which 10 served as the intact group—healthy rats that did not undergo any intervention and whose results represented the physiological baseline. Chronic heart failure was experimentally induced in the remaining 30 animals.

CHF in rats was modeled by administering three intraperitoneal injections of hydrochloric adrenaline at a dose of 0.3 mg/kg body weight, spaced 48 h apart [22]. The used dosage induces metabolic damage to the myocardium, leading to focal necrosis and corresponding microcirculatory disturbances [24]. The development of heart failure was confirmed using echocardiography with a Doppler ultrasound system for small animals (S12-4, Philips CX50, Holland, The Netherlands) on days 14 and 30 after the last adrenaline injection. Additionally, histological examination of the myocardium was conducted on day 30 [22].

### 2.3. Experimental Design

On the day following the last adrenaline injection, the 30 animals were randomly divided into three groups (*n* = 10) using a random number generator (RandStuff.ru). The animals were placed in ventilated boxes (50 L) for 40 min daily. During five consecutive days, molecular hydrogen was administered into the boxes using a hydrogen generator “Sputnik-3” (Shandong Saikesaisi, Hydrogen Energy Co., Ltd., Heze, Shandong, China), delivering a gas mixture containing 2% H_2_, 96% air, and 21% O_2_ to allow the animals to breathe freely. Gas concentrations within the boxes were monitored with a portable analyser for explosive and toxic gases and vapors, “Hydrogen (H_2_)” (“Signal-4,” Moscow, Russia). Rats in the second group inhaled the hydrogen–air mixture (2% H_2_) for 40 min on the day after modeling of heart failure, after which they were exposed to ambient air in the box for the next four days. The third group (control group) animals were placed in a ventilated box filled with atmospheric air for 40 min daily over five days following the last injection.

### 2.4. Materials and Stages of the Study

Blood samples and tissue microcirculation parameters were examined on days 1, 3, 7, and 14 after the last adrenaline injection. Blood for analysis was obtained from the sublingual vein using standard methods. The macroscopic and microscopic examination of the rat hearts was performed on day 14 after the final adrenaline injection.

In our research group, in which rats developed CHF and were then treated with H_2_, all animals were included in the analysis. There were no exceptions to the data, as all rats successfully underwent heart failure simulation and treatment without serious complications or losses. During the allocation of rats to different groups, the researchers were aware of each animal’s group membership. Throughout the experiment, the individuals who administered treatment and monitored the animals were unaware of the group assignments in order to minimize bias. Additionally, the data analysis was conducted in a blind manner to ensure the objectivity of the results.

### 2.5. Research Methods

#### 2.5.1. Assessment of Peripheral Blood Flow

The peripheral blood flow in vivo was evaluated using laser Doppler flowmetry (LDF) with the “Lazma-ST” device (Passport IABZh.94.1111.011 REP, LLC “NPP Lazma”, Moscow, Russia). The “Lazma-ST” system includes: the “Lazma-D” analyzer with a fixed fiber-optic probe for microcirculation assessment and coenzyme fluorescence amplitude measurements; the “Lazma-TEST” module for conducting temperature and electrical stimulation tests; and dedicated software.

For the procedure, the rat was placed in a transparent, ventilated box that limited movement, with the tail protruding through an opening in the rear wall and fixed at the level of the body with adhesive tape. The fiber-optic sensor of the “Lazma-ST” device was positioned perpendicular to and fixed on the base of the tail. The animal was acclimated to the environment for 5 min before measurements. Laser Doppler flowmetry was then performed, with a recording duration of 15 min for each LDF trace.

LDF reflects microcirculatory changes in approximately 1 mm^3^ of tissue. Since the reflection from static tissue structures has a constant frequency, flowmetry is based on analyzing the Doppler shift in the reflected signal originating from mobile elements, namely erythrocytes. This shift depends on their hematocrit, the number of functional capillaries, and the linear velocity of blood cells within the microcirculatory system. Consequently, the baseline tissue perfusion (TP) can be expressed by the following equation:

TP = K × N (erythrocytes) × v (average),

where TP—tissue perfusion index (signal amplitude in perfusion units (PU));

K—the proportionality coefficient (mm × Volts) (assumed to be 1);

N (erythrocytes)—the number of erythrocytes within the irradiated tissue volume;

v (average)—the average linear velocity of erythrocytes within the irradiated tissue volume.

The mean values of perfusion parameters (M_TP_) provide an overall assessment of microcirculatory status. Thus, M_TP_—the arithmetic means of the microcirculatory tissue perfusion index (PM), measured in perfusion units (PU)—reflects the total number of erythrocytes across all levels of the microcirculatory system within the irradiated tissue volume during the recording period.

To evaluate the degree of blood flow modulation, the following parameters were assessed:

The flux level σ (Standard Deviation)—the root means square deviation of blood flow amplitude fluctuations relative to M_TP_, measured in perfusion units (PU), reflecting the extent of temporal blood flow modulation resulting from vasomotions driven by active and passive regulatory mechanisms.

Cv—the coefficient of variation in blood flow oscillations, defined as the ratio of σ to MPM and expressed as a percentage (%).

Microcirculatory regulation is carried out by neural and humoral mechanisms that modulate the vessel diameter of the microcirculatory bed according to tissue needs.

Periodic nonlinear oscillations of the LDF signals are characterized by five frequency intervals, each corresponding to different types of physiological regulatory mechanisms affecting microcirculation. Endothelial (0.005–0.021 Hz), neurogenic (0.021–0.052 Hz), and myogenic (0.052–0.145 Hz) oscillations are associated with local (peripheral) control mechanisms of vasodilation. Conversely, cardiac (0.6–2 Hz) and respiratory (0.145–0.6 Hz) oscillations are driven by central mechanisms influencing tissue perfusion [25]. To determine the type of regulatory mechanism, the amplitude-frequency spectrum (AFS) of perfusion oscillations was calculated using wavelet transformation, performed with the software of the LAKK series device, version 3.2.0.538 (LLC “NPP LAZMA” Moscow, Russia).

The predominance of active or passive regulation mechanisms of microcirculation was assessed using the fluxogenic index (FI), calculated according to the following formula:

FI = (A_e_ + A_n_ + A_m_)/(A_c_ + A_r_),

where A_e_—the amplitude of endothelial oscillations (PU);

A_m_—the amplitude of myogenic oscillations (PU);

A_n_—the amplitude of neurogenic oscillations (PU);

A_c_—the amplitude of cardiac oscillations (PU);

A_r_—the amplitude of respiratory oscillations (PU).

#### 2.5.2. Assessment of Tissue Metabolism Status

The status of tissue energy metabolism was evaluated based on the quantitative changes in biomarkers—coenzymes nicotinamide adenine dinucleotide (NADH) and flavin adenine dinucleotide (FAD). The levels of reduced NADH and oxidized FAD, as well as the metabolic index (MI), were determined using laser fluorescence spectroscopy (LFS) with the “LAZMA-D” analyzer (LLC “NPP LAZMA”, Moscow, Russia).

The fluorescence amplitude was recorded using an ultraviolet (UV) spectral filter at the excitation wavelength of 365 nm, corresponding to the peak absorption of mitochondrial NADH, with the emission spectrum measured between 460 and 470 nm, since the fluorescence intensity of cytoplasmic NADH is known to be very low. The fluorescence amplitude of FAD was recorded using a blue spectral filter (Blue) at the excitation wavelength of 450 nm, with the emission spectrum measured between 510 and 520 nm.

For a comprehensive assessment of the relationship between tissue metabolism and microcirculation, the metabolic index (MI) was analyzed, calculated using the following formula:

MI = M_TP_/(A_NADH_ + A_FAD_),

where M_TP_—the mean value of the microcirculatory tissue perfusion index,

A_NADH_—the amplitude of NADH coenzyme fluorescence,

A_FAD_—the amplitude of FAD coenzyme fluorescence.

#### 2.5.3. Examination of the Adaptive Capacity of Tissue Metabolism

Temperature tests were conducted to assess the adaptive capacity of tissues by cooling the tissue to 10 °C to reduce microcirculatory and metabolic activity, and heating it to 35 °C to increase activity, using the “LAZMA-TEST” device. The study was conducted in a room with a constant ambient temperature of 22 ± 1 °C. The skin temperature at the base of the rat’s tail during baseline testing was 31.2 ± 0.4 °C. Temperature was monitored using an integrated thermocouple and maintained at this level throughout the experiment. The cold test recording duration was 1 min after cooling the tissue to 10 °C to prevent the onset of cold-induced vasodilation. The measurement period for the heated tissue was 4 min after raising the temperature of the test area to 35 °C.

The assessment was performed by calculating the coefficients:

C (^−^)—the skin’s adaptive activity coefficient during cooling to 10 °C.

C (^−^) = 1 − (M_TP baseline_ − M_TP 10_)/M_TP baseline_,

where M_TP baseline_—the mean value of the microcirculatory tissue perfusion index during the baseline test (without temperature exposure)

M_TP 10_—the mean value of the microcirculatory tissue perfusion index during cooling of the tissue to 10 °C.

C (^+^)—the coefficient of skin adaptive activity during heating to 35 °C.

C (^+^) = 1 − (M_TP 35_ − M_TP baseline_)/M_TP baseline_,

where M_TP 35_—the mean value of the microcirculatory tissue perfusion index during heating of the tissue to 35 °C

M_TP baseline_—the mean value of the microcirculatory tissue perfusion index during the baseline test (without temperature exposure.

A reduction in the values of C (^+^) and C (^−^) relative to baseline indicates an increased energy demand and a decreased reserve of the tissue’s adaptive capacity.

#### 2.5.4. Lipid Peroxidation

The activity of lipid peroxidation (LPO) processes was assessed by changes in the content of dienic conjugates (DC), trienic conjugates (TC), and Schiff bases (SB) in plasma, using spectrophotometry of lipid extract phases on the SF-2000 spectrophotometer (ZAO “OKB Spectrum”, Saint Petersburg, Russia).

Optical density (E) of the heptane and isopropanol phases was evaluated based on the absorption of monochromatic light through a 1 cm optical path. The results were expressed in relative units, calculated as the ratio of the absorption at specific wavelengths—DC at 233 nm (E_233_), TC at 278 nm (E_278_), and SB at 400 nm (E_400_)—to the optical control measurement at 220 nm (E_220_).

#### 2.5.5. Assessment of Antioxidative Enzyme Activities

The levels of antioxidant-related parameters were measured in blood plasma. To determine the activity of catalase (CAT), a modified method based on Maehly and Chance (1954) was employed [26]. This method is based on measuring the rate of hydrogen peroxide (H_2_O_2_) decomposition by the enzyme catalase. In the procedure, 1.0 mL of a 30 µM H_2_O_2_ solution was added to the sample, and the reduction in its concentration was monitored spectrophotometrically at a wavelength of 240 nm. Plasma samples were diluted to the required concentration, and measurements were performed at fixed time intervals (1 min) to determine enzymatic activity, which was expressed in units per volume of plasma.

#### 2.5.6. Measurement of Malondialdehyde (MDA) Levels in Cardiac Tissue

To assess the level of oxidative stress in the myocardium MDA concentration was measured. The analysis was performed using a spectrophotometric assay based on the thiobarbituric acid reactive substances (TBARS) method in myocardial tissue homogenates. Rat cardiac tissues were homogenized in ice-cold phosphate-buffered physiological saline. TBARS levels were assessed following the method of Bar-Or et al. [27]. Tissue homogenates diluted with phosphate buffer were incubated on ice for 10 min. An acid reagent containing HCl, TCA, and TBA was added, and samples were boiled for 15 min, then cooled to 4 °C. After centrifugation, absorbance was read at 532 nm. TBARS concentration was calculated using an extinction coefficient of 1.56 × 10^5^ M^−1^cm^−1^.

#### 2.5.7. Macroscopic and Microscopic Examination of the Heart

Due to the limited informativeness of ejection fraction (EF) in rats, which does not always accurately reflect overall systolic function and ventricular remodeling [28], a histological examination was performed for a comprehensive assessment of myocardial injury. Although echocardiography is prioritized for its relatively low-cost, non-invasive assessment of changes associated with cardiomyopathies, researchers encounter specific challenges due to rapid heart rates and small animal size. These difficulties necessitate the development of additional standardization procedures for data acquisition and analysis [29]. In addition to histological analysis, myocardial mass was measured in all groups. This parameter objectively confirms the presence of structural remodeling of the myocardium underlying the development of heart failure. Meanwhile, histological examination provides direct morphological evidence of the pathological processes responsible for cardiac dysfunction. In this study, histological data were used to elucidate the mechanisms of remodeling and potential compensatory changes in the myocardium induced by hydrogen therapy.

The animals’ hearts were harvested on the 14th day after the last adrenaline injection, weighed, and their relative mass was calculated. According to the experimental protocol, animals were humanely euthanized by administering an overdose of anesthesia (PENBITAL, Bioveta a.s., Ivanovice na Hane, Czech Republic) to minimize pain, suffering, and distress during the collection of cardiac tissue for histological analysis.

Myocardial fixation for histological examination was performed in a 10% neutral buffered formalin solution for 4 days. The myocardium was embedded in paraffin blocks and sectioned into 10 serial slices, each 5–7 μm thick. The sections were stained with hematoxylin and eosin (H&E).

Morphometric analysis was performed using a transmitted light microscope Vizo-101 (LOMO, Saint Petersburg, Russia). The analysis focused on the number of functioning capillaries per 1 mm^2^ of myocardial tissue section (n/mm^2^), capillary diameter (μm), cardiomyocyte diameter (μm), cardiomyocyte nuclear area (μm^2^), edema area relative to the section area (%), and area of hemorrhage relative to the section area (%).

### 2.6. Statistical Analysis

Comparative data analysis was performed using the STATISTICA v. 12 software package (StatSoft, Inc., Tulsa, OK, USA). The Shapiro–Wilk test was employed to assess data normality, considering its reliability for small to medium sample sizes. Upon confirmation of normal distribution, parametric statistical tests were applied, including Student’s *t*-test with Bonferroni correction and one-way ANOVA, followed by post hoc comparisons using Duncan’s test.

Sample size estimation was performed using the classical formula for comparing two independent means (two-sided *t*-test), with a significance level of *α* = 0.05 and a statistical power of 80% (1 − *β* = 0.80). Anticipated between-group difference (Δ) for the parameter of interest was 4 units, with a standard deviation (*σ*) of 2.9. Using *Z*-values of 1.96 (for *α* = 0.05) and 0.84 (for *β* = 0.20), the calculated sample size per group was

$$n=2\times \left(\frac{Z_{1}-\alpha }{2+Z_{1}-\beta }\right)\times \frac{\sigma^{2}}{{∆}^{2}}=8.24$$

*n* = 2 × (1.96 + 0.84)^2^ × 2.9^2^/4^2^ = 2 × 7.84 × 0.53 = 8.24

To account for multiple comparisons (Bonferroni correction) and the potential exclusion of animals, the target sample size was increased to 10 animals per group. Descriptive statistics, including mean values and standard error of the mean (M ± SEM), were calculated following Student’s methodology. Statistical significance was considered at a threshold of *p* < 0.05.

## 3. Results

### 3.1. Molecular Hydrogen Enhances Tissue Perfusion by Strengthening the Active Mechanisms Regulating the Tension of the Vascular Smooth Muscle Layer in Microcirculatory Vessels

**Figure 1:**
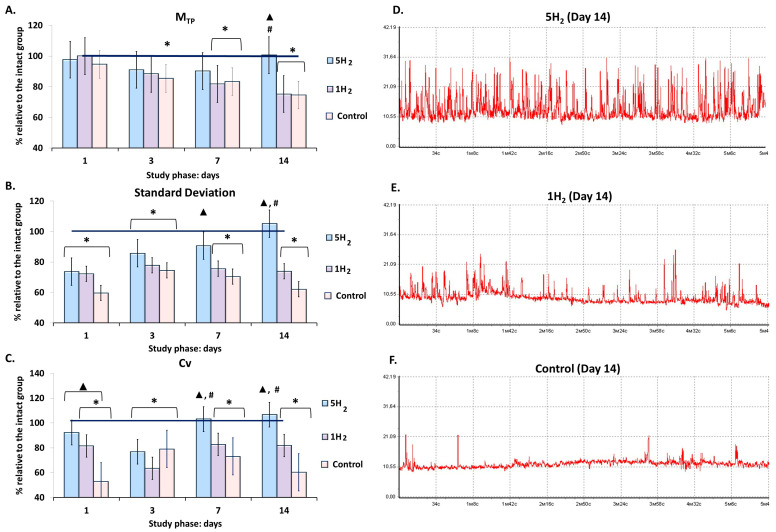
Dynamics of baseline tissue perfusion parameters in rats at early stages of chronic heart failure (CHF) development and after single and multiple treatments with molecular hydrogen. (**A**) Histogram of perfusion index (M_TP_) in the studied groups. (**B**) Histogram of amplitude deviation of blood flow oscillations (σ, standard deviation, flux) in the studied groups. (**C**) Histogram of coefficient of variation in blood flow oscillations (Cv) in the studied groups. (**D**) Representative laser Doppler flowmetry (LDF) trace of a rat from the multiple hydrogen treatment group (5H_2_) on day 14. (**E**) Representative LDF trace of a rat from the single hydrogen treatment group (1H_2_) on day 14. (**F**) Representative LDF trace of a rat from the control group on day 14. Data are shown as mean ± SEM. *—statistically significant difference compared to the intact group (*p* < 0.05); ▲—statistically significant difference compared to the control group (*p* < 0.05); #—statistically significant difference between multiple and single hydrogen treatment groups (*p* < 0.05).

Laser Doppler flowmetry of microcirculation systems revealed that, in the control group, the baseline tissue perfusion (M_TP_) gradually decreased relative to the baseline values of the intact group, starting from 3rd day after the last adrenaline injection. This finding indicates a progressive depletion of tissue blood flow. The flux level (σ) exhibited its maximum reduction of 40% on day 1; by day 3, a trend toward recovery was noted. However, subsequently, σ decreased again, reaching a level 38% below physiological norms by day 14. A similar pattern was observed for the coefficient of variation (Cv), which showed a maximum decrease of 48% on day 1 (Table 1, Figure 1). The observed changes in σ and Cv values indicate a suppression of adaptive modulation of tissue blood flow, likely resulting from diminished activity of vasomotor control mechanisms in the CHF model.

In the group subjected to multiple H_2_ inhalations, the M_TP_ exceeded the control group values by 35% on day 14. In contrast, the single-inhalation H_2_ group showed a statistically significant decrease in this parameter starting from day 7 relative to the intact group, with the difference reaching 33% by day 14 compared to the repeated inhalation group (Table 1, Figure 1).

The flux level and coefficient of variation increased in both groups during the early phases of the study. In the single-inhalation H_2_ group, these parameters were higher than in the control group but declined from day 7 onward, and by the end of the experiment, the flux level was 26% lower and the coefficient of variation was 18% lower than the values observed in the intact group on day 14. An increase in the degree of temporal modulation of blood flow and the activity of vasomotor regulatory mechanisms was evident in the multiple-inhalation group, with these parameters remaining within the range characteristic of the intact animals and exceeding the control group values by 69% for flux level and 77% for the coefficient of variation on day 14 of the study (Table 1, Figure 1).

The frequency (F, Hz) characteristic of the low-frequency (LF) power spectrum allows for the identification of the type of regulatory mechanism, since different mechanisms operate within specific frequency ranges. The amplitude values (A, relative units) of endothelial-origin flux oscillations (Ae) are associated with the secretory activity of the inner layer of large arteriole walls and allow for assessment of the degree of influence exerted by a metabolically humoral mechanism on the lumen of microvascular channels. Amplitudes of neurogenic (An) origin reflect the activity of sympathetic (adrenergic) regulation, while myogenic (Am) amplitudes indicate the dynamics of ion (Ca^2+^, K^+^) diffusion across the membranes of smooth muscle cells in arterioles, pre-capillary sphincters, and capillary sphincters. Thus, changes in Ae, An, and Am directly reflect factors influencing the vascular wall musculature in microcirculatory channels. Heartbeat (Ac) and respiratory (Ar) oscillation amplitudes characterize the dynamics of perfusion and vascular pressure in the microcirculatory vessels, depending on fluctuations in the pressure gradient in large proximal vessels and the suction effect of the chest during inspiration. Consequently, these parameters reflect the contribution of central regulatory mechanisms [30,31].

**Figure 2:**
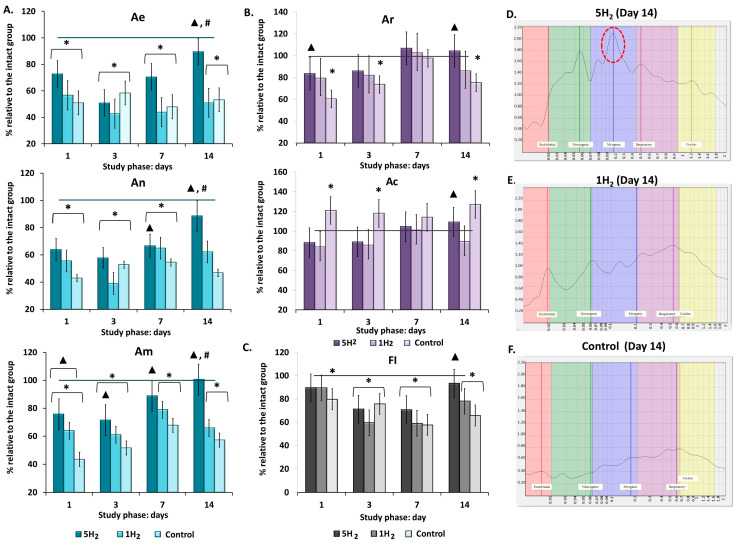
Dynamics of amplitude-frequency characteristics (AFC) of tissue perfusion in rats during early stages of chronic heart failure (CHF) development and after single and multiple treatments with molecular hydrogen. (**A**) Histograms of active regulatory mechanisms in the studied groups (endothelial (Ae), neurogenic (An), myogenic (Am) oscillations). (**B**) Histograms of passive regulatory mechanisms in the studied groups (respiratory (Ar), cardiac (Ac) oscillations). (**C**) Histogram of flux motion index (FI). (**D**) Laser Doppler flowmetry (LDF) spectrum of a rat from the multiple hydrogen treatment group (5H_2_) on day 14 (Am is the dominant of the study (highlighted in red)). (**E**) LDF spectrum of a rat from the single hydrogen treatment group (1H2) on day 14. (**F**) LDF spectrum of a rat from the control group on day 14. Data are presented as mean ± SEM. *—statistically significant difference compared to the intact group (*p* < 0.05); ▲—statistically significant difference compared to the control group (*p* < 0.05); #—statistically significant difference between multiple and single hydrogen treatment groups (*p* < 0.05).

Wavelet spectrum analysis of the control group revealed the predominance of central components of tissue perfusion regulation and the development of ischemic stagnation. A reduction in endothelial (Ae) by 47.39%, neurogenic (An) by 46.71%, and myogenic (Am) amplitudes by 42.85% was observed compared to healthy animals. Conversely, an increase in cardiac (Ac) amplitude was associated with a 52.25% decrease in the fluxogenic index (FI) (Table 2, Figure 2A,B,F). Thus, maintaining adequate tissue metabolism is achieved through increased cardiac workload and elevated central arterial pressure.

Repeated inhalations of H_2_ during the early stages of heart failure development facilitated the mobilization of local vasodilation regulation pathways and prevented cumulative myocardial remodeling via two mechanisms: reducing afterload on the heart and increasing blood flow within the myocardial microcirculation. By day 14, all regulatory parameters returned to the levels observed in the intact group. Among the amplitudes of local factors, Am was predominant on days 3, 7, and 14 of the study (Table 2, Figure 2A–C). A single H_2_ administration also contributed to an increase in peripheral regulation; however, this effect was less pronounced than in the group with repeated H_2_ inhalations (Table 2, Figure 2A,D,E). Additionally, the fluxogenic index (FI) was 31% below the normal range (Table 2, Figure 2B), indicating a predominant influence of cardiopulmonary factors through the elevation of the arterio-venous pressure gradient.

### 3.2. Molecular Hydrogen Enhances Tissue Energetic Aerobic Metabolism and Promotes the Engagement of Adaptive Mechanisms to Sustain It

**Figure 3:**
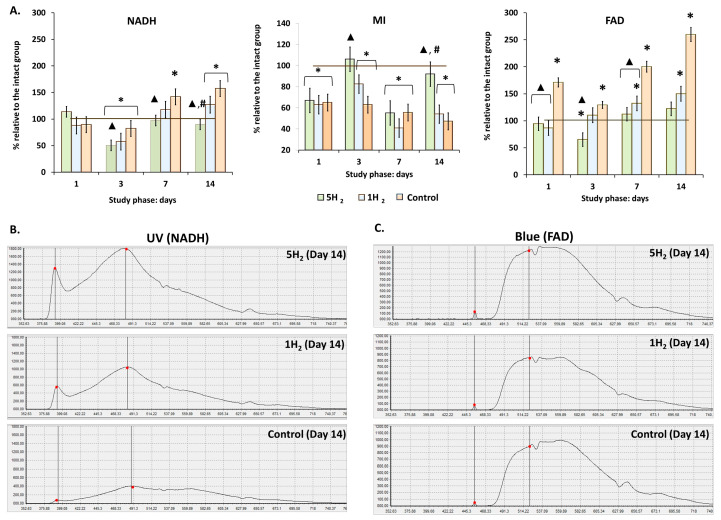
Dynamics of tissue metabolism in rats at early stages of experimental chronic heart failure (CHF) development and after single and multiple treatments with molecular hydrogen. (**A**) Histograms of intracellular metabolism indicators. (**B**) Laser Doppler flowmetry (LDF) trace of NADH fluorescence (UV laser excitation). (**C**) LDF trace of FAD fluorescence (Blue laser excitation). Data are presented as mean ± SEM. *—statistically significant difference compared to the intact group (*p* < 0.05); ▲—statistically significant difference compared to the control group (*p* < 0.05); #—statistically significant difference between multiple and single hydrogen treatment groups (*p* < 0.05).

Following the confirmation of H_2_′s role in increasing nutritive blood flow through the activation of local regulatory factors, the metabolic state of tissues was assessed. Quantitative evaluation of endogenous fluorescence intensity of respiratory chain coenzymes allows insight into cellular metabolic activity [32]. To assess the effects of inhalation of 2% H_2_ on intracellular metabolism, we measured the fluorescence levels of reduced NADH and oxidized FAD. Repeated administration of high doses of adrenaline significantly increased the fluorescence levels of these coenzymes over the subsequent 14 days. At the same time, a gradual decrease in the metabolic index (MI) was observed, with the minimum values (47% of physiological norm) recorded on day 14 (Table 3, Figure 3). Our results indicated that repeated administration of H_2_ significantly reduced NADH and FAD levels compared to the control group and the group receiving a single H_2_ inhalation. Conversely, the metabolic index (MI) increased and was 73.68% higher than control values by day 14. Collectively, these findings indicate that inhalation of 2% H_2_ effectively supports aerobic mitochondrial respiration, preventing excessive ROS production and the development of oxidative stress in rats during the early stages of heart failure progression (Figure 3).

**Figure 4:**
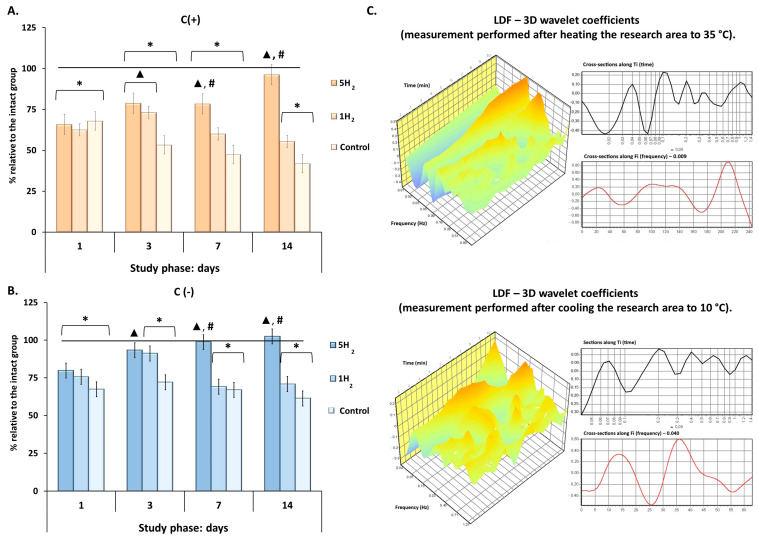
Dynamics of the adaptation coefficient of the microcirculatory-tissue system in rats during early stages of experimental chronic heart failure (CHF) development and after single and multiple treatments with molecular hydrogen. (**A**) Histogram of the adaptation coefficient during tissue heating to 35 °C. (**B**) Histogram of the adaptation coefficient during tissue cooling to 10 °C. (**C**) Laser Doppler Flowmetry (LDF)—3D wavelet coefficients of a rat receiving repeated H_2_ treatment on day 14 of the study. Data are presented as mean ± SEM. *—statistically significant difference compared to the intact group (*p* < 0.05); ▲—statistically significant difference compared to the control group (*p* < 0.05); #—statistically significant difference between multiple and single hydrogen treatment groups (*p* < 0.05).

For further evaluation of tissue metabolism, an experiment assessing the adaptive capacity was conducted by recording the adaptation coefficient of the microcirculatory-tissue system in rats during cooling (up to 10 °C) and heating (up to 35 °C) of the tissue (Figure 4). Analysis of the temperature test results revealed significant differences between the experimental groups. Our results demonstrated a significant increase in the adaptation coefficient during both heating (C (^+^)) and cooling (C (^−^)) in the group receiving repeated H_2_ inhalations compared to the control group, as well as compared to the group with a single H_2_ inhalation, starting from day 7 (Figure 4).

In summary, these results indicate that prolonged hyperadrenergemia provokes a subsequent sharp slowdown of energy-dependent metabolic processes and depletion of adaptive reserves. Additionally, repeated H_2_ inhalations administered in the early stages after pathological exposure may mitigate this imbalance, aiding in the recovery of metabolism to physiological levels and preventing damage to tissues most sensitive to hypoxia.

### 3.3. Molecular Hydrogen Reduces the Activity of Lipid Peroxidation Processes

An increase in reactive oxygen species (ROS) and the activation of lipid peroxidation processes significantly contribute to microcirculatory impairments. This impairment leads to a local oxygen deficiency, which further stimulates ROS production and the generation of lipid peroxidation products, thereby establishing a self-perpetuating vicious cycle that exacerbates oxidative damage and microvascular dysfunction.

To elucidate the antioxidant mechanism of H_2_, this study assessed the levels of LPO products and catalase activity in rat blood plasma. These measurements provided insights into the potential protective effects of H_2_ against lipid membrane damage during the early stages of chronic heart failure (CHF) development.

The results demonstrated that there were no significant changes in the levels of dienic conjugates (DC) and trienic conjugates (TC) across all study groups (Table 4). This phenomenon can be attributed to the pronounced instability of these compounds, leading to their rapid conversion into highly reactive but more stable end products of LPO.

Schiff bases (SB) are stable end products formed during lipid peroxidation. In the control group, SB levels were elevated at all stages of the study relative to the values of the intact group. Peaks occurred on days 3 and 14 after the last adrenaline injection. These findings indicate persistent oxidative stress and ongoing membrane lipid damage in this group. In contrast, both H_2_-treated groups exhibited an increase in SB levels only on the first day. From day 3 onward, the levels gradually decreased and, by the end of the study, returned to the physiological normative range. However, repeated H_2_ administration had a more pronounced effect: on day 3, SB levels in the repeated inhalation group were 41.95% lower than those in the single-exposure group, with the difference increasing to 42.93% by day 14 (Table 4).

Furthermore, the results indicated an enhancement of antioxidant defense mechanisms in the H_2_-treated groups. Specifically, catalase activity increased by a factor of 2.11 at 14 days in the group subjected to repeated H_2_ inhalation, while the single-exposure group demonstrated a return to baseline enzymatic activity. In contrast, the control group exhibited a significant decline of approximately 2.5-fold in catalase activity at 14 days relative to the intact level (Table 4). These findings suggest that H_2_ inhibits the formation of LPO products and enhances endogenous antioxidant enzyme activity. This mitigates cumulative lipid peroxidation in cellular membranes, thereby preventing oxidative tissue damage.

### 3.4. The Early Administration of Molecular Hydrogen During the Development CHF Contributes to the Preservation of Microcirculation and Myocardial Morphology at Physiological Levels and Limiting the Processes of Lipoperoxidation of the Heart

Histological evaluation of myocardial alterations following prolonged sympathoadrenal stimulation and subsequent hydrogen administration was performed. Myocardial tissue samples were harvested and subjected to hematoxylin and eosin (H&E) staining on day 14 post-final adrenaline administration to assess structural changes at the tissue level.

**Figure 5:**
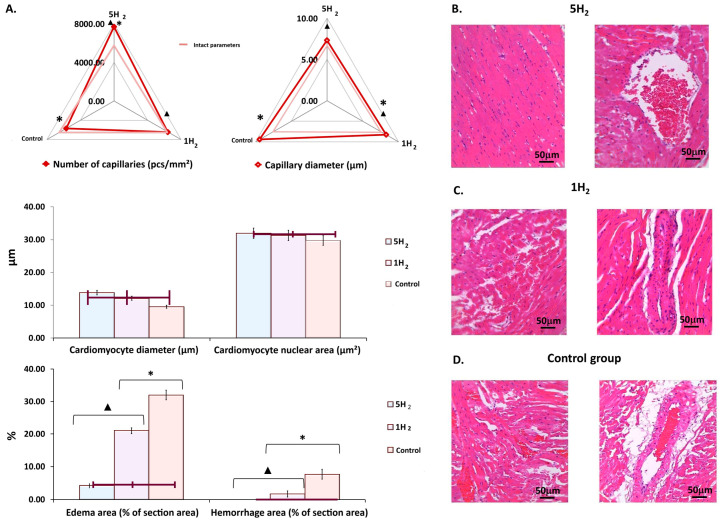
Morphological changes in rat myocardium on day 14 after the last injection of adrenaline in the studied groups. (**A**) Diagrams showing changes in morphometric parameters of the myocardium. (**B**) Myocardium of a rat after multiple applications of molecular hydrogen. (**C**) Myocardium of a rat after a single application of molecular hydrogen. (**D**) Myocardium of a control rat. Scale bar: 50 µm (magnification: UV. approx. ×7, total magnification: ×20). Quantitative analysis was performed on 10 fields of view. Data are presented as mean ± SEM. *—statistically significant difference compared to the intact group (*p* < 0.05); ▲—statistically significant difference compared to the control group (*p* < 0.05).

Significant microcirculatory disturbances were observed in the myocardium of the control group, including large red thrombi in arterioles, sludging in capillaries, marked venular congestion, and numerous hemorrhages. Additionally, severe interstitial and perivascular edema was noted, alongside multiple foci of cardiomyocyte hypercontraction, cardiomyocytes exhibiting uneven cytoplasmic staining, and areas of muscle fiber fragmentation (Figure 5D). Notably, similar but less pronounced changes were observed in the group receiving a single administration of hydrogen (Figure 5B). Conversely, in the myocardium of rats receiving repeated hydrogen therapy, the extent of perivascular and interstitial edema was significantly reduced relative to the other two groups. Areas of myofiber fragmentation were absent, with only isolated foci of cardiomyocyte hypercontraction and some cardiomyocytes displaying uneven cytoplasmic staining. Hemorrhages and thrombi within arterioles were not observed; however, some capillaries exhibited sludging of erythrocytes, and moderate venular congestion was present in certain venules (Figure 5A). These findings suggest a protective effect of repeated hydrogen administration against microvascular and myocardial structural disturbances associated with the experimental condition.

Morphometric assessment of the myocardium in the control group demonstrated a significant reduction of 18.72% in capillary density, accompanied by a 13.02% increase in capillary luminal diameter. Additionally, there was an 18.65% hypertrophy of cardiomyocytes, and the area of perivascular edema and hemorrhagic lesions was markedly increased by 7.8-fold (Figure 5A,D).

A comparative morphometric analysis of myocardial tissue following single versus multiple hydrogen treatments revealed an increase in capillary number by approximately 13% and 35%, respectively, compared to the control group, when averaged across the entire myocardium. Concurrently, a reduction in capillary lumen diameter was observed—by 12% after a single exposure and by 32% with repeated hydrogen administration, relative to controls (Figure 5A,B). Additionally, repetitive hydrogen therapy was associated with an 11% decrease in cardiomyocyte diameter compared to the control group. These structural changes were accompanied by a significant reduction in the areas of edema and hemorrhages within the myocardium (Figure 5A,B).

Macroscopic examination of the myocardium in the control group rats on day 14 post-modeling of CHF revealed a 29% increase in heart weight compared to the physiological baseline (intact group). Hydrogen (H_2_) treatment was associated with a reduction in heart weight, and repeated H_2_ administration produced statistically significant differences compared to the control group (Table 2). Additionally, the MDA level in the myocardial tissue was evaluated as an indicator of lipid peroxidation. The results demonstrated that MDA levels were significantly reduced following H_2_ treatment in comparison to rats subjected to experimental CHF modeling without H_2_ therapy (Table 5).

Therefore, the significant microcirculatory disturbances observed in response to repeated administration of high doses of catecholamines into the peripheral circulation likely triggered mechanisms associated with oxidative stress, inflammation, and apoptosis [33]. This, in turn, exacerbated the emerging hemodynamic and perfusion-metabolic disturbances, depleting the body’s adaptive reserves, thereby contributing to myocardial dysfunction and the development of chronic heart failure. Repeated hydrogen therapy effectively mitigated the previously described disruptions by attenuating oxidative stress and preserving microcirculatory integrity, thereby ensuring adequate perfusion and metabolic homeostasis essential for myocardial function.

## 4. Discussion

This study elucidates the antioxidant properties of molecular hydrogen, which regulate microcirculatory mechanisms and tissue metabolism, thereby mitigating myocardial ischemia–reperfusion injury associated with heart failure. To evaluate the therapeutic effect of hydrogen treatment, we compared the parameters of rats that received single or repeated hydrogen inhalation with those of animals that did not undergo any therapeutic interventions during the initial stages of heart failure development. The dynamics of all obtained LDF data indicate the progression of a stagnant microcirculatory failure, corresponding to moderate (II) and severe (III) stages. The moderate stage is characterized by a 10–25% reduction in tissue blood flow, while the severe stage involves a 25–40% decrease within the control group [34]. One of the mechanisms contributing to vascular dysfunction may be the increased production of superoxide anion radicals, as these reactive oxygen species rapidly react with nitric oxide (NO) to form peroxynitrite, which induces vasoconstriction by elevating vascular resistance [35].

Repeated administration of hydrogen inhalation promoted an increase in tissue perfusion, attributed to vasodilation of microcirculatory vessels. This finding aligns with results observed in human studies where consumption of hydrogen-rich water enhanced flow-mediated vasodilatory responses [36]. Based on the amplitude-frequency characteristics obtained in our study, we can discuss the mechanisms by which molecular hydrogen modulates microhemodynamics. An increase in the endothelial activity index (Ae) indicates enhanced secretory function of the endothelium and elevated synthesis of nitric oxide (NO), a potent vasodilator that reduces the tone of small arteries and large arterioles [30,31]. This effect supports the maintenance of blood pressure balance between central vessels and the microcirculatory hemodynamic segment, thereby increasing both volumetric and velocity parameters of tissue blood flow. Furthermore, the sustained increase in the amplitude of neurogenic oscillations (An) throughout the experiment suggests a reduction in the tone of arteriovenous shunts and small arterioles due to diminished sympathetic adrenergic influence. Finally, the increase in the amplitude of myogenic oscillations (Am) reflects restoration of the receptor apparatus of smooth muscle cells in terminal arterioles and precapillary sphincters, optimizing the function of membrane ion channels regulating K^+^ and Ca^2+^ exchange, which contributes to maintaining capillary microcirculation at physiological levels [31,32].

These findings complement our previous research on the effects of H_2_ on erythrocytes—cells responsible for oxygen transport in blood [22]. Specifically, H_2_ inhalation reduced erythrocyte aggregation, and increased cell surface charge, ATP, and 2,3-diphosphoglycerate (2,3-DPG) levels in rats during CHF development. Conversely, CHF was associated with increased aggregation and decreased surface charge and metabolic activity. The improvement of erythrocyte parameters with H_2_ therapy during CHF enhances blood rheology and reduces deoxygenation in capillaries, thereby supporting the restoration of blood oxygen-carrying capacity. Thus, the previously observed enhancement of blood oxygen transport capacity is complemented by the present findings, which demonstrate improved microcirculatory function resulting from the activation of peripheral vasoregulation mechanisms by molecular hydrogen. This contributes to the maintenance of homeostasis and prevents tissue hypoxia.

Moreover, the increase in central regulation parameters of perfusion (Ar and Ac) in the control group indicates the engagement of an ergotropic compensatory mechanism, which amplifies the influence of cardio-respiratory factors to enhance the arterio-venous pressure gradient, thereby promoting increased blood flow to the microcirculatory vessels. However, the augmentation of pulse wave amplitude facilitates enhanced vascular wall injury within the microcirculation, leading to increased vasoconstriction and further reduction in tissue perfusion. Activation of tone-regulating factors at the microcirculatory vessel level by molecular hydrogen results in relaxation of the smooth muscle cells in the vessel wall, consequently decreasing overall vascular resistance. This process obviates the need to elevate central arterial pressure. As a result, there is no increase in afterload on the heart, which also contributes to maintaining adequate myocardial perfusion.

The use of hydrogen inhalation also influenced the state of tissue energy metabolism. In the control animals, the levels of NADH and FAD were elevated, while ATP levels were significantly below the physiological norm, indicating dysfunction of mitochondrial respiratory chain complexes I and II, which leads to decreased ATP synthesis [37]. Repeated administration of hydrogen helped maintain aerobic mitochondrial respiration at physiological levels. Numerous studies have demonstrated that mitochondrial complexes I and II are the primary sources of ROS production within mitochondria [38,39]. ROS can be generated either indirectly through complex II (the electron transfer reverse mechanism), where, in the presence of high succinate concentrations, electrons from the reduced ubiquinone pool are transferred to complex I, resulting in the production of pathological levels of ROS [40,41], or directly, by transferring electrons from excess FADH2 to oxygen and additionally through amplified reverse electron transport (RET), which elevates the generation of ROS [42,43]. In turn, highly reactive ROS damage mitochondrial membranes, impairing ATP synthesis.

Specific properties of H_2_, such as its small size, neutral charge, and nonpolar nature, facilitate rapid transmembrane diffusion, allowing it to reach intracellular organelles within one minute and modulate their functions [44]. The observed reduction in NADH and FAD concentrations, along with an increased MI ratio in the groups treated with hydrogen inhalation, can be explained by H_2_′s antioxidant activity—corroborated in our study by decreased levels of lipid peroxidation products both in the blood plasma and in the myocardium. Furthermore, experimental data from the literature confirm that H_2_ selectively scavenges not only hydroxyl radicals and peroxynitrite but also mitochondrial ROS, notably by inhibiting superoxide production at complex I [45,46]. Direct inhibition of NADPH oxidase expression and the reduction in mitochondrial damage lead to suppression of ROS production and the subsequent downregulation of downstream signaling pathways such as ERK1/2, p38, and JNK, thereby contributing to the protective effects of H_2_. [47]. Therefore, based on the literature and our findings of enhanced aerobic metabolic processes, it can be hypothesized that H_2_, by inhibiting free radical oxidation, modulates mitochondrial bioenergetics and consequently confers cardioprotection.

The antioxidant effect of H_2_ was confirmed by evaluating lipid peroxidation activity through changes in levels of lipid peroxidation products in blood plasma, concomitant with increased catalase activity. A significant reduction in Schiff bases—the most toxic end products of lipid peroxidation—in rat plasma was observed as early as day 3 following H_2_ treatment and persisted throughout the subsequent stages of the study. Schiff bases are highly reactive, capable of polymerization, polycondensation, and generating pathological intermolecular adducts that accumulate in chronic diseases and aging, thereby impairing tissue functional activity [48,49]. Additionally, an elevation in catalase activity was detected by day 3 of H_2_ exposure in our study.

Regarding the additional effects of molecular hydrogen, several studies have demonstrated that H_2_ can activate Nrf2 and promote its translocation to the nucleus, thereby enhancing the transcription of antioxidant enzymes such as catalase (CAT) and glutathione peroxidase 1 (GPX1) [50]. Furthermore, it has been demonstrated that overexpression of CAT in the heart effectively reduces intracellular ROS levels, improves mitochondrial structural integrity, and promotes the maintenance of normal cardiac function [51]. Our study demonstrated that H_2_ inhalation reduces oxidative myocardial injury during the early stages of CHF modeling.

Histological and morphometric examination of the myocardium demonstrated that the significant antioxidant and metabolic effects of H_2_ in the early stages of CHF development—particularly with repeated administration—facilitated trophic alterations in myocardial microcirculation. Specifically, these effects were associated with increased nutritive blood flow and venous drainage, a reduction in tissue edema, and preservation of the structural integrity of the majority of cardiomyocytes. Consequently, these changes help prevent the progression to irreversible morphological and functional myocardial damage, thereby potentially reducing the need for prolonged, expensive therapeutic interventions.

Considering the role of hemodynamic parameters in the progression of [52], our data show that H_2_ inhalation has beneficial effects. It improves metabolic and oxidative markers in the blood vessels. It also helps normalize the regulation of microvascular tone. These effects may help prevent or limit heart muscle remodeling in CHF.

Thus, the application H_2_ in the management of CHF may be regarded as a safe and efficacious prophylactic and therapeutic approach. However, our study has several limitations that must be acknowledged. Although inhalation administration of H_2_ is the most physiologically relevant and safe method, accurately determining the optimal individual dose for each subject proves to be challenging. Moreover, the 14-day duration of the study precludes the assessment of the long-term efficacy and safety of H_2_ therapy, including whether the initial beneficial effects will persist or reach a plateau, thereby sustaining myocardial functional activity. There remains a concern regarding the potential disturbance of the oxidative-antioxidative balance with prolonged H_2_ administration. Finally, the possibility of organismal adaptation or tolerance to repeated H_2_ exposure warrants further investigation.

Currently, there are no established global standards regarding the dosage and duration of H_2_ administration for preventive and therapeutic purposes. Additionally, this study was limited to a model of sympathetic-adrenal stimulation in rats, and other models of CHF have not been investigated. Future studies will be necessary to validate and expand upon these findings. To address these issues, we plan to extend the duration of H_2_ exposure and conduct long-term studies to investigate the mechanisms of action of molecular hydrogen. Additionally, we aim to develop protocols for the use of H_2_ as an active antioxidant with the goal of preventing the development of CHF. Furthermore, it is important to note that numerous studies (over 2000 publications) have demonstrated the non-toxicity of molecular hydrogen across a wide range of concentrations. However, to establish safety within a specific disease model, it is necessary to analyze biochemical and histological markers of toxicity.

## 5. Conclusions

In conclusion, this study demonstrates the trophic role of H_2_ during the early stages of chronic heart failure development. H_2_ restores nutritional blood flow, tissue perfusion, and energy metabolism to physiologically appropriate levels, thereby preventing tissue hypoxia and oxidative stress. These effects help preserve myocardial contractile function. Consequently, H_2_ may serve as a promising prophylactic strategy in cases of prolonged sympathetic-adrenal stimulation.

## Figures and Tables

**Table 1:** Baseline tissue perfusion parameters in rats during early stages of experimental CHF development, following single and multiple treatments with molecular hydrogen (M ± SEM).

Study Parameter	Group	Day 1	Day 3	Day 7	Day 14
M_TP_ (PU)	Control	11.88 ± 1.66	10.72 ± 0.63 *	10.46 ± 1.32 *	9.36 ± 1.12 *
	5H_2_	12.26 ± 1.20	11.43 ± 0.73	11.33 ± 1.17	12.63 ± 0.84 ^▲#^
	1H_2_	12.56 ± 1.36	11.10 ± 0.86	10.27 ± 1.52 *	9.45 ± 1.01 *^#^
Standard deviation (σ) (PU)	Control	2.77 ± 0.23 *	3.46 ± 0.26 *	3.27 ± 0.31 *	2.88 ± 0.35 *
	5H_2_	3.42 ± 0.13 *^▲^	3.98 ± 0.44	3.97 ± 0.31 ^▲^	4.88 ± 0.19 ^▲#^
	1H_2_	3.35 ± 0.28 *^▲^	3.61 ± 0.19 *	3.53 ± 0.29 *	3.43 ± 0.11 *^▲#^
Cv (%)	Control	19.55 ± 5.68 *	29.19 ± 4.06	26.98 ± 2.31 *	22.27 ± 1.97 *
	5H_2_	34.09 ± 3.33 ^▲^	28.38 ± 2.06 *	38.07 ± 3.34 ^▲#^	39.38 ± 3.02 ^▲#^
	1H_2_	30.09 ± 3.07 *^▲^	23.42 ± 2.82 *	30.55 ± 3.13 ^#^	30.24 ± 1.04 *^▲#^

Note: M_TP_—the baseline tissue perfusion; Cv—the coefficient of variation. Values for intact animals: M_TP_—12.56 ± 0.83 perfusion units; Standard deviation—4.64 ± 0.77 perfusion units; Cv—36.92 ± 5.50%. “*”—statistically significant difference compared to intact group, *p* < 0.05; “▲”—statistically significant difference in the experimental group compared to control, *p* < 0.05; “#”—statistically significant difference between multiple and single molecular hydrogen treatment groups, *p* < 0.05.

**Table 2:** Dynamics of amplitude-frequency characteristics of tissue perfusion in rats during early stages of experimental CHF development, following single and multiple treatments with molecular hydrogen (M ± SEM).

Study Parameter	Group	Day 1	Day 3	Day 7	Day 14
Ae (PU)	Control	0.88 ± 0.32 *	1.01 ± 0.12 *	0.83 ± 0.05 *	0.92 ± 0.24 *
	5H_2_	1.26 ± 0.15 *	0.88 ± 0.18 *	1.22 ± 0.17 *^▲#^	1.55 ± 0.26 ^▲#^
	1H_2_	0.98 ± 0.17 *	0.74 ± 0.16 *	0.76 ± 0.07 *#	0.88 ± 0.33 *^#^
An (PU)	Control	0.78 ± 0.19 *	0.96 ± 0.16 *	0.99 ± 0.05 *	0.85 ± 0.11 *
	5H_2_	1.16 ± 0.15 *	1.05 ± 0.21 *	1.21 ± 0.13 *^▲^	1.61 ± 0.25 ^▲#^
	1H_2_	1.01 ± 0.08 *	0.71 ± 0.17 *	1.18 ± 0.16 *^▲^	1.13 ± 0.29 *^#^
Am (PU)	Control	0.70 ± 0.19 *	0.83 ± 0.13 *	1.09 ± 0.15 *	0.92 ± 0.09 *
	5H_2_	1.22 ± 0.11 *^▲^	1.15 ± 0.18 *^▲^	1.43 ± 0.15 ^▲^	1.62 ± 0.08 ^▲#^
	1H_2_	1.03 ± 0.11 *^▲^	0.98 ± 0.22 *	1.17 ± 0.13 *	1.06 ± 0.11 *^#^
Ar (PU)	Control	0.73 ± 0.22 *	0.89 ± 0.13 *	1.18 ± 0.19	0.91 ± 0.05 *
	5H_2_	1.01 ± 0.04 *^▲^	1.04 ± 0.16	1.29 ± 0.13	1.26 ± 0.06 ^▲^
	1H_2_	0.96 ± 0.08 *	0.99 ± 0.13	1.24 ± 0.13	1.04 ± 0.12
Ac (PU)	Control	1.35 ± 0.12 *	1.31 ± 0.11 *	1.27 ± 0.09	1.41 ± 0.13 *
	5H_2_	0.98 ± 0.09	0.904 ± 0.16	1.16 ± 0.14	1.21 ± 0.14 ^▲^
	1H_2_	0.93 ± 0.08	0.95 ± 0.13	1.12 ± 0.11	0.99 ± 0.11
FI (PU)	Control	1.13 ± 0.12 *	1.27 ± 0.31 *	1.18 ± 0.47 *	1.16 ± 0.21 *
	5H_2_	1.81 ± 0.27	1.58 ± 0.14 *	1.57 ± 0.18 *	1.94 ± 0.24 ^▲^
	1H_2_	1.83 ± 0.19	1.32 ± 0.11 *	1.31 ± 0.09 *	1.51 ± 0.21 *

Note: Ae—amplitude of endothelial oscillations; An—amplitude of neurogenic oscillations, Am—amplitude of myogenic oscillations, Ar—amplitude of respiratory oscillations, Ac—amplitude of respiratory oscillations, FI—fluxomyometry index. Values for intact animals: Ae—1.73 ± 0.22 perfusion units; An—1.82 ± 0.26 perfusion units; Am—1.61 ± 0.24 perfusion units; Ar—1.21 ± 0.14 perfusion units; Ac—1.11 ± 0.11 perfusion units; FI—2.22 ± 0.21 perfusion units. “*”—statistically significant difference compared to intact group, *p* < 0.05; “▲”—statistically significant difference in the experimental group compared to control, *p* < 0.05; “#”—statistically significant difference between multiple and single molecular hydrogen treatment groups, *p* < 0.05.

**Table 3:** Dynamics of tissue metabolism parameters in rats during early stages of experimental CHF development following single and multiple treatments with molecular hydrogen (M ± SEM).

Study Parameter	Group	Day 1	Day 3	Day 7	Day 14
NADH	Control	1.22 ± 0.25	1.07 ± 0.11 *	2.10 ± 0.09 *	2.33 ± 0.38 *
	5H_2_	1.88 ± 0.40	0.79 ± 0.16 *^▲^	1.44 ± 0.26 ^▲^	1.33 ± 0.38 ^▲#^
	1H_2_	1.05 ± 0.33	0.85 ± 0.13 *	1.74 ± 0.34	1.88 ± 0.12 *^#^
FAD	Control	13.89 ± 1.48 *	10.50 ± 0.46 *	16.26 ± 2.42 *	21.07 ± 4.33 *
	5H_2_	7.66 ± 0.63 ^▲^	5.29 ± 0.46 *^▲#^	9.10 ± 2.11 ^▲^	9.91 ± 2.51 ^▲^
	1H_2_	7.03 ± 1.33 ^▲^	8.95 ± 2.31 #	10.73 ± 1.01 *^▲^	12.18 ± 2.48 *^▲^
MI	Control	3.14 ± 0.49 *	3.04 ± 0.52 *	2.68 ± 0.71 *	2.28 ± 0.76 *
	5H_2_	3.24 ± 0.66 *	5.12 ± 0.96 ^▲^	2.66 ± 0.84 *	3.96 ± 0.44 ^▲#^
	1H_2_	3.04 ± 0.76 *	3.98 ± 0.42 *^#^	1.57 ± 0.21 *	2.61 ± 0.33 *^#^

Note: MI—the metabolic index. Values for intact animals: NADH—1.48 ±0.27; FAD—8.12 ± 0.30; MI—4.83 ± 0.45%. “*”—statistically significant difference compared to intact group, *p* < 0.05; “▲”—statistically significant difference in the experimental group compared to control, *p* < 0.05; “#”—statistically significant difference between multiple and single molecular hydrogen treatment groups, *p* < 0.05.

**Table 4:** Dynamics of plasma lipid peroxidation products in rats during early stages of experimental chronic heart failure (CHF), following single and multiple hydrogen treatments (M ± SEM).

Study Parameter	Group	Day 1	Day 3	Day 7	Day 14
DC (relative units)	Control	0.18 ± 0.01	0.17 ± 0.03	0.15 ± 0.01	0.19 ± 0.01
	5H_2_	0.19 ± 0.01	0.21 ± 0.01 *	0.16 ± 0.01	0.21 ± 0.02 *
	1H_2_	0.20 ± 0.01	0.18 ± 0.01	0.16 ± 0.01	0.18 ± 0.02
TC (relative units)	Control	0.07 ± 0.01	0.09 ± 0.02	0.06 ± 0.01	0.07 ± 0.01
	5H_2_	0.07 ± 0.02	0.12 ± 0.01 *	0.08 ± 0.01	0.08 ± 0.01
	1H_2_	0.07 ± 0.02	0.12 ± 0.01 *	0.08 ± 0.01	0.08 ± 0.01
SB (relative units)	Control	5.05 ± 0.83 *	6.56 ± 1.75 *	4.04 ± 0.84	6.55 ± 0.73 *
	5H_2_	5.54 ± 0.92 *	2.38 ± 0.25 ^▲#^	2.57 ± 0.75 ^▲^	2.14 ± 0.64 ^▲#^
	1H_2_	6.47 ± 1.73 *	4.10 ± 0.93 ^▲#^	2.52 ± 0.88 ^▲^	3.75 ± 1.12 ^▲#^
Catalase activity (U/gHb·min)	Control	0.55 ± 0.13 *	0.62 ± 0.140 *	0.63 ± 0.27 *	0.73 ± 0.33
	5H_2_	0.49 ± 0.12 *	0.95 ± 0.15 ^▲^	0.69 ± 0.18 *	1.54 ± 0.21 *^▲^
	1H_2_	0.80 ± 0.19 *	0.79 ± 0.07 *	0.74 ± 0.29 *	1.27 ± 0.13 ^▲^

Note: DC—diene conjugates; TC—triene conjugates; SB—Schiff bases. Values for intact animals: DC—0.17 ± 0.01 relative units; TC—0.07 ± 0.02 relative units; SB—3.01 ± 0.65 relative units, Catalase activity—1.14 ± 0.14 (U/gHb·min). “*”—statistically significant difference compared to intact group, *p* < 0.05; “▲”—statistically significant difference in the experimental group compared to control, *p* < 0.05; “#”—statistically significant difference between multiple and single molecular hydrogen treatment groups, *p* < 0.05.

**Table 5:** Heart weight and myocardial MDA concentration on day 14 post-modeling of chronic heart failure (CHF), following single and multiple hydrogen treatments (M ± SEM).

Group	Heart Mass/Body Weight, %	MDA, nmol/L
Control	0.66 ± 0.02	1.95 ± 0.10 *
5H_2_	0.59 ± 0.04 ^▲^	1.24 ± 0.08 ^▲^
1H_2_	0.64 ± 0.02	1.47 ± 0.08 *^▲#^

Note: Values of intact animals: heart mass/body weight—0.505 ± 0.031%; MDA—1.30 ± 0.06 nmol/L. *—statistically significant difference compared to the intact group (*p* < 0.05); ▲—statistically significant difference compared to the control group (*p* < 0.05); #—statistically significant difference between multiple and single hydrogen treatment groups (*p* < 0.05).

## Data Availability

All data generated or analyzed during this study are included within this published article.
